# Response of Plant and Soil N, P, and N:P Stoichiometry to N Addition in China: A Meta-Analysis

**DOI:** 10.3390/plants12112104

**Published:** 2023-05-25

**Authors:** Shuifei Chen, Wenwen Zhang, Xiaomin Ge, Xiao Zheng, Xu Zhou, Hui Ding, Aiguo Zhang

**Affiliations:** Nanjing Institute of Environmental Sciences, State Environmental Protection Scientific Observation and Research Station for Ecological Environment of Wuyi Mountains, State Environmental Protection Key Laboratory on Biosafety, Ministry of Ecology and Environment of China, Nanjing 210042, China; chenshuifei@163.com (S.C.);

**Keywords:** N deposition, terrestrial ecosystem, ecological stoichiometry, meta-analysis

## Abstract

Nitrogen (N) and phosphorus (P) are key elements essential for plant growth and development. Due to fertilizer application, rapid urbanization, and fossil fuel combustion, nitrogen deposition has reached relatively high levels in China. However, there is still uncertainty regarding the response of N:P stoichiometry in plants and soil to N deposition across different ecosystems. Therefore, a meta-analysis was conducted using 845 observations from 75 studies to evaluate the response of plant and soil N and P concentrations and N to P ratios across various ecosystems to N addition. The analysis revealed that N concentration and N:P stoichiometry in plants and soil increased under N addition, while P concentration in plants and soil decreased on average. Furthermore, the magnitude of these responses was related to the N input rate and experimental duration. Finally, the effects of N addition on N concentration, P concentration, and N:P in terrestrial ecosystems would alter their allocation patterns, depending on relevant climate factors such as mean annual temperature and mean annual precipitation. This study highlights the ecological impact of N addition on the biogeochemical cycling of major elements (N and P) in terrestrial ecosystems in China. These findings are necessary for improving our understanding of the characteristics of plant ecological stoichiometry and helping to plan measures for increasing N deposition.

## 1. Introduction

With the rapid development of socioeconomic conditions, the widespread use of agricultural fertilizers, and the increase in fossil fuel combustion, atmospheric nitrogen deposition nitrogen has been considered as a key factor causing global change [1,2]. China has become a hotspot of nitrogen (N) deposition after Western Europe and North America [1,3]. The N deposition rate in several regions in China has reached or even exceeded the peak N deposition rate observed in Europe during the 1980s [4]. In comparison to the 1980s, China’s NH3 emissions have doubled (13.7 Tg N yr^−1^), and NOx emissions have quadrupled (6.0 Tg N yr^−1^) at the beginning of this century [4]. Although some studies have found that the N deposition rate has stagnated in China [5], it is still relatively high and has received researchers’ attention [6]. Taking into account the response of plant and soil chemical elements to atmospheric nitrogen deposition, we have simulated nitrogen deposition through nitrogen addition experiments. Numerous empirical studies have shown that N addition accelerates the rate of N mineralization and nitrification in soil, thereby increasing the soil available N and changing the N and P concentrations ([N] and [P]) in plants [7,8,9]. Consequently, the dominant plant species within a community tends to actively respond to N deposition, leading to its rapid growth, which affects the community structure and biodiversity [10,11]. Meanwhile, N and P, important components of plant cell structure, play critical roles in producing and transforming proteins and nucleic acids [12]. Generally, the N and P content of leaves, as an index of soil nutrient limitation, affects plant growth strategies [13]. On other hand, the response of different ecological compartments to N addition was different in different ecosystems [14]. Therefore, a better understanding of the response of the N and P stoichiometry (N:P) in plant and soil to N addition is essential for predicting the impact of increased N deposition on understanding the adaptation of various plants in terrestrial ecosystems in China.

N and P are widespread limiting elements in terrestrial ecosystems [15]. Researchers reported that the nutrient stoichiometry in the ecosystem compartments (plant vegetative organs, soil, or litter) could be a good indicator predicting plant nutrient restriction [16,17]. For example, when N:P of ecosystem compartments < 14, limited N restricts plant growth; when N:P > 16, limited P slows plant growth; and when N:P is between 14 and 16, it indicates a colimitation of N and P [16]. However, the use of N:P to judge the nutrient limitation to plant growth remains controversial. Several studies have reported that the N:P of ecosystem compartments <10 and >20 can be used as a criteria for judging plant growth restriction by N and P, respectively [18]. Based on this theory, it was assumed that the [N] of ecological compartments rises due to N addition. Subsequently, to maintain a specific quantitative relationship between limited N and limited P in the ecosystem compartments, [P] also increases while N:P remains unchanged.

Many studies on simulated N deposition have been carried out in China to examine the response of [N], [P], and N:P in the terrestrial ecosystem to N addition [19,20,21]. However, the response varies among the ecosystems in these studies. For instance, in a subtropical forest, the leaf [N] did not respond to N addition, but [P] increased, resulting in a decrease in the N:P of leaves [22]. In grassland, although N addition reduced the N resorption efficiency (NRE), it significantly promoted an increase in leaf [N], [P], and N:P [23]. A similar response was found in N and P allocation patterns in leaves in a wetland [24]. These differences may be due to the differences in terrestrial ecosystems and species with different strategies in nutrient enrichment in the nutrient distribution mode of the vegetative organs in the plant [22,25]. Therefore, it can be speculated that the effects of N addition on [N], [P], and N:P may vary depending on the ecosystem types and the climate factors.

The response of [N], [P], and N:P in plants and soil to N addition may also be related to the experimental duration and the N input rate [11,26]. In generally, N addition may increase the soil available N, change the soil C:N, affect the microbial N limit, and promote microbial participation in soil mineralization and nitrification, thereby increasing the soil available N and promoting plant absorption [27,28,29]. However, the levels of N addition in the experiment might have exceeded the levels of N deposition, which may lead to N leaching and acidification in the soil, further affecting the soil microbial biomass [26,30]. These all might lead to the loss of a large amount of nitrogen compounds from the soil. Therefore, continuous N addition may change [N] and [P] in the terrestrial ecosystem. Studies have found that constant N deposition has transformed the growth of plants in terrestrial ecosystems from an N limitation to a P limitation [19,31].

Furthermore, studies have indicated that the response of [N], [P], and N:P in plants and soil to N addition varies with the ecosystem compartments [21,26]. In a forest ecosystem, the increase in [N] and [P] and C:N:P stoichiometry in soil was more sensitive than that in leaves under N addition [32]. In a grassland, leaves N:P demonstrated a significant response to N addition via the effects on soil nutrients, soil stoichiometry, and plant community composition, while root N:P stoichiometry changed via the impact on soil nutrients and stoichiometric ratios [20]. Therefore, the effects of N addition on [N], [P], and N:P in the terrestrial ecosystems may vary with the ecosystem compartments. Considering the possible opposite results of N addition, a quantitative evaluation that unifies the results of each experiment can help to understand the [N], [P], N:P of plant and soil responses to N addition.

A recent meta-analysis addressed the response of leaf [N], [P], and N:P to N addition in the grasslands of northern China [2]. However, the spatial scale was small, and only the response of a single ecological compartment to N addition was analyzed. In addition, the effect of N addition on the various ecosystem compartments under different ecosystems has not been considered. Therefore, the present study conducted a meta-analysis based on 845 observations from 75 studies, which was substantially more than the previous meta-analysis, thus allowing us to examine multiple ecosystem compartments simultaneously. The objectives of this study were to analyze (1) the differences in the effects of N addition on [N], [P], and N:P among different ecosystem compartments and (2) the role of N input rate, experimental duration, ecosystem type, and geographical factors in regulating the responses of [N], [P], and N:P. The findings will be important for understanding and modelling the effects of N deposition on the biogeochemical cycles of the terrestrial ecosystems in China [33].

## 2. Results

### 2.1. The Effects of N Addition on N, P, and N:P

**Figure 1:**
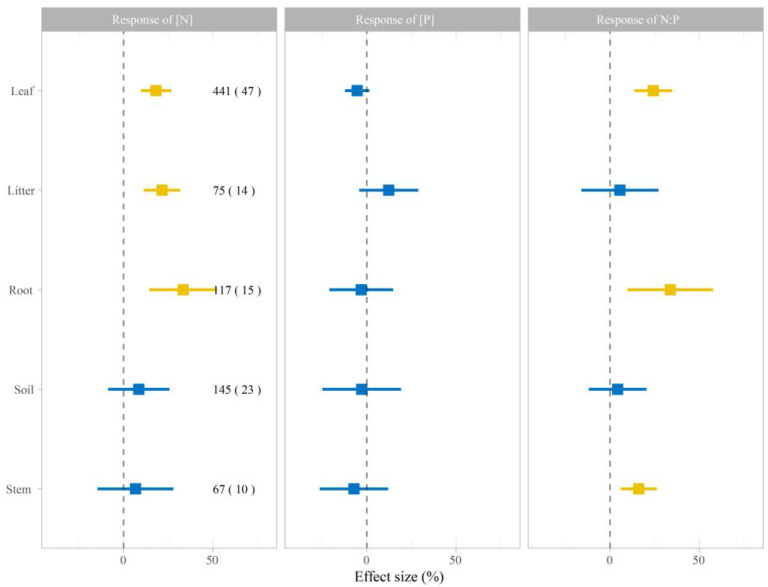
Effects of nitrogen (N) addition on the [N], [P], and N:P of leaves, litter, roots, soil, and stems. The values shown are mean ± 95% confidence intervals (CI). Values inside and outside the parentheses represent the number of observations and studies, respectively. Blue and yellow quadrates represent variables with 95% CI that do not overlap with zero (*p* < 0.05) and that overlap with zero (*P* > 0.05), respectively.

The response of [N], [P], and N:P to N addition were different among ecosystem compartments in plant and soil. The [N] in leaves, litter, and roots, on average, significantly increased with N addition by 18.6% (95% confidence interval [CI], 9.67–26.65%), 21.52% (95% CI, 11.36–31.79%), and 33.38% (95% CI, 14.42–52.33%), respectively. However, no significant increase in [N] of soil (*p* = 0.84) and stem (*p* = 0.68) was observed (Figure 1). The effects of N addition on [P] in the five ecosystem compartments were not significant (*p* > 0.05; Figure 1). Meanwhile, N addition significantly increased the N:P stoichiometry of leaves (95% CI, 13.68–34.80%), roots (95% CI, 9.74–57.73%), and stems (95% CI, 5.99–26.18%) (Figure 1). However, the responses of soil (95% CI, −11.92–20.43%) and litter (95% CI, −16.06–27.12%) were not significant to N addition (Figure 1).

### 2.2. Influence of N Input Rate and Experimental Duration on the Effects Size

**Figure 2:**
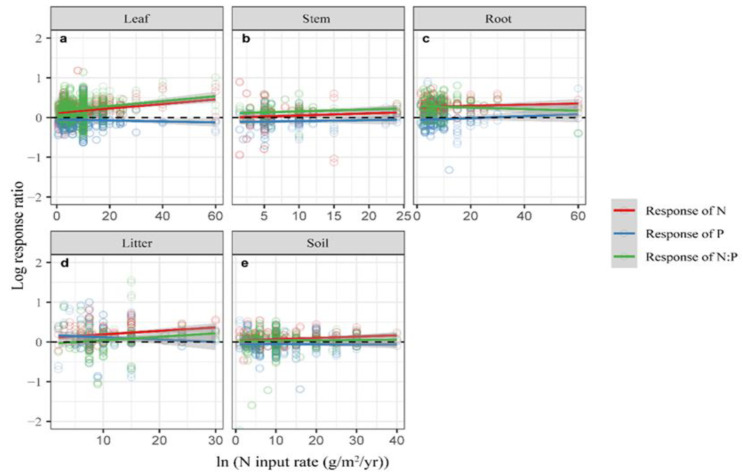
Relationship between nitrogen input rate and natural logarithm response ratio (ln*RR*) of [N], [P], and N:P in leaves (**a**), stems (**b**), roots (**c**), litter (**d**), and soil (**e**) under N addition. Open circles represent the observations of a specific element. Colored lines are fitted by partial linear regression, with shaded areas representing the 95% confidence intervals. *p*-values are shown in Table 1.

**Figure 3:**
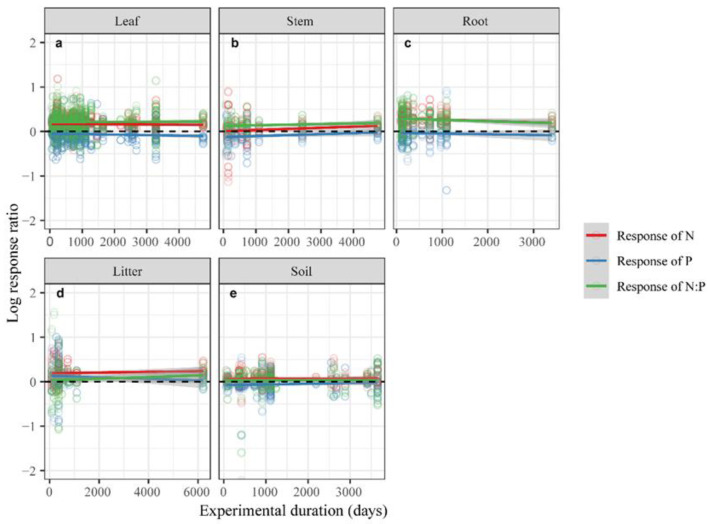
Relationship between experimental duration and natural logarithm response ratio (ln*RR*) of [N], [P], and N:P in leaves (**a**), stems (**b**), roots (**c**), litter (**d**), and soil (**e**) under N addition. Open circles represent the observations of a specific element. Colored lines are fitted by partial linear regressions, with shaded areas representing the 95% confidence intervals. *p*-values are shown in Table 1.

Partial linear regression was employed to examine the effects of N and *ED* on [N], [P], and N:P in plants and soil, under N addition (Table 1, Figure 2 and Figure 3). [N] of the leaves (*p* < 0.01), litter (*p* < 0.01), and soil (*p* = 0.01) showed a significant positive response to N addition, with the increase in N input (Figure 2, Table 1). However, no significant linear relationship was found between N addition and [P] (Table 1). Only the N:P of the leaves (*p* < 0.01) showed a significant positive response to N input, under N addition (Table 1). However, the N:P of the leaves was negative with an increase of the experimental duration (*p* = 0.02, Figure 3). Furthermore, with an increasing experimental duration, the ln*RR* for [P] in leaves increased significantly (Figure 3; Table 1). These results suggested that the response of ecosystem compartments to N addition should be changed with N input rate and experimental duration.

### 2.3. Influence of Ecosystem Types and Geographical Factors on Effect Size

**Figure 4:**
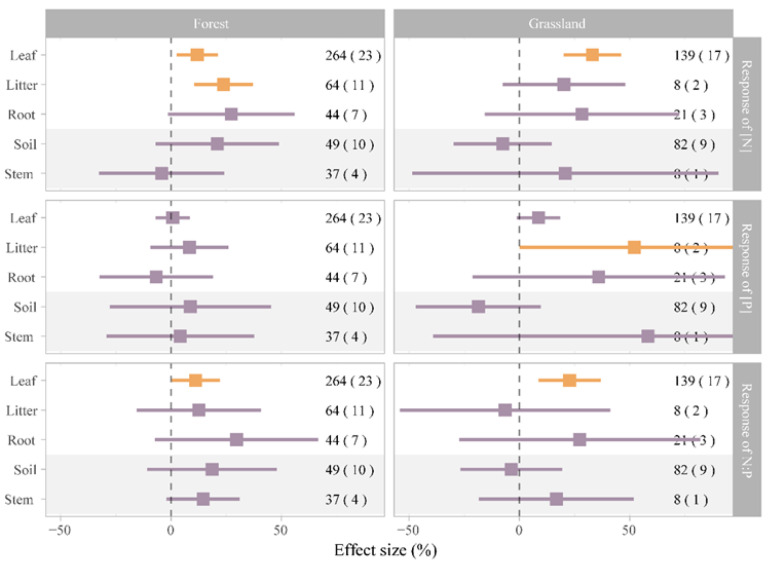
Effects of nitrogen (N) addition on [N], [P], and N:P of leaves, litter, roots, soil, and stems in a forest ecosystem and a grassland ecosystem. Orange and purple quadrates represent variables with 95% confidence intervals that do not overlap with zero (*p* < 0.05) and that overlap with zero (*P* > 0.05), respectively.

**Figure 5:**
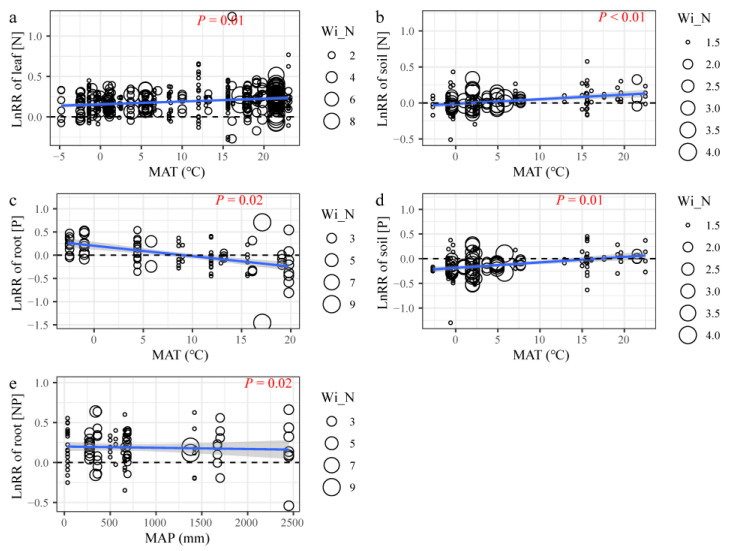
Effects of mean annual temperature (MAT °C) on natural logarithm response ratio (ln*RR*) of [N] and N:P of the leaves (**a**), soil (**b**), roots (**c**), and soil (**d**), and effects of mean annual precipitation (MAP mm) on ln*RR* of N:P (**e**), under N addition. MAT or MAP with a significant impact on [N], [P], and N:P is displayed. Wi_N represents the weights of the research.

The response of [N], [P], and N:P in plants and soil to N addition did not change with the ecosystem types (forest, grassland, wetland, and pot; Table 2), indicating a consistent response across ecosystems in China. However, when we performed analysis separately, the results revealed that the mean effects of N addition varied among the ecosystem compartments in different ecosystem types (Figure 4). In four ecosystems, the response of [N] and N:P in leaves to N addition were significant, whereas that of soil [N] and N:P was not significant (Figure 4 and Figures S1 and S2). However, the response of [P] of plants and soil to N addition was not significant in four ecosystems (Figure 4 and Figures S1 and S2). To figure out geographical factors that affect effects of N addition, we extract mean annual precipitation (MAT) and mean annual precipitation (MAP) of four ecosystems. The impact of MAT on ln*RR* of leaf [N], soil [N], and soil [P] differed significantly (Figure 5a,b), indicating an inconsistency in the response of [N] of leaves and soil to N addition under different climate conditions in China. The inconsistency with response to N addition was also found in the N:P stoichiometry of roots (Figure 5e).

## 3. Discussion

This meta-analysis based on 845 observations of 75 studies is the first to examine the effects of N addition on the [N], [P], and N:P of plants and soil across various climates and ecosystems in China. This study also explored the effects of *ED*, N, geographical factors, and ecosystem types on the [N], [P], and N:P of different ecosystem compartments. The findings provide new insights into the relationships between N addition and [N], [P] and N:P stoichiometry in the compartments of diverse ecosystems of China. The [N] and N:P in plants and soil responded positively to N addition in the terrestrial ecosystems; however, the effect was insignificant in specific ecosystem compartments, such as stems, soil, and litter. In addition, N addition had no effect on [P]. The effect size of N addition depended on N input rate and experimental duration, and the effects of N input rate on [N], [P], and N:P were consistent. Moreover, changes in geographical factors, such as MAT and MAP, influenced the natural logarithm response ratios.

### 3.1. Effects of N Addition on [N], [P], and N:P Ratio of Plant and Soil in China

This meta-analysis revealed a positive response of [N] and N:P in leaves and roots to N addition in China (Figure 1), consistent with the previous meta-analyses by Su et al. [2] and Xu et al. [34]. Earlier studies have also shown that N addition increased both the aboveground and belowground [N] of plants, and that leaves and roots responded synergistically to N addition. Generally, with an increase in N addition, the soil available N increases, promoting N absorption by plants and increasing the N concentration of leaves and roots [26]. The present meta-analysis found that N addition improved the [N] and N:P of soil in China; however, on average, the effect on [P] of soil was negative but not significant (Figure 1). These findings imply that soil [P] is associated with plant growth, reducing the [P] in the soil. On the other hand, eluviation in soil decreased [P] in soil. Meanwhile, on average, the response of [P] of different ecosystem compartments decreased with N addition; however, this was not significant, except for litter (Figure 1). Available P is derived via weathering of parent material in terrestrial ecosystems but not from biological processes [35]. Once weathering stops, the available P is obtained mainly via the decomposition and recovery of P from the litter [36]. The input of external nitrogen accelerates phosphatase synthesis and promotes available phosphorus [37,38]. Therefore, an increase in [P] of litter was seen. In addition, experiments have suggested that soil P availability might decrease with increased N addition in the secondary forest due to land use causing soil acidification, indicating that limited P was universal in China [38,39]. Many primary forests have been deforested in China during the past several centuries [40], probably decreasing [P] in the terrestrial ecosystem caused by land use. This might be a main reason that in the context of nitrogen addition with soil acidification, the [P] of terrestrial ecosystems decreased.

Recently, a meta-analysis showed that changes in plant C-N-P stoichiometry caused by N addition were significantly affected by pH on a global scale [14]. Soil pH affects soil nutrient dynamics by altering microbial activity or elemental forms, which in turn affects the ability of plants to absorb and utilize soil nutrients [14]. Furthermore, a separate analysis revealed that [N] and N:P in the leaves of plants in forest, grassland, wetland, and pot ecosystems responded significantly to the N input rate, whereas [N] and N:P in the other compartments, such as roots, soil and stems, had no response to N addition (Figure 5 and Figures S2 and S3). The differences in the effects observed for the different compartments via the meta-analysis may be due to the difference in sample size among the individual studies. The growth rate hypothesis might explain the more significant response of [P] to N addition in grassland than in forest [41]. Usually, in a forest ecosystem, tall trees are K-strategists with lower maximal specific growth rates, whereas grassland and herbs with short lifecycles are r-strategists with features of fast growth [42]. Species with high growth rates have higher P requirements to ensure the rapid and efficient synthesis of RNA and the accumulation of biomass [41,43].

### 3.2. Main Factors Influencing the Response of These under N Addition

This meta-analysis revealed that the response of [N] in plants and soil to the N input rate was positive; however, [P] in plants and soil decreased with increasing experimental duration (Figure 3 and Figure 4, Table 1). These results suggested that N input and experimental duration influenced [P] in plants and soil, indicating critical points of N input for the ecosystems [44]. In addition, N input exceeding the ecosystem N demand will reduce phosphatase activity and P mobility in soil and inhibit productivity [45,46]. Therefore, in production practice, the input rate of nitrogen fertilizer and the duration would affect the N and P uptake by plants, which in turn affects plant growth. Studies have also shown that N addition causes soil acidification, thus causing a decrease in phosphatase activity and inhibiting soil microbial growth, which absorbs a large amount of phosphorus from the soil as an energy source for its respiration [42,46]. Therefore, the ability to hold P in the soil would decrease.

**Figure 6:**
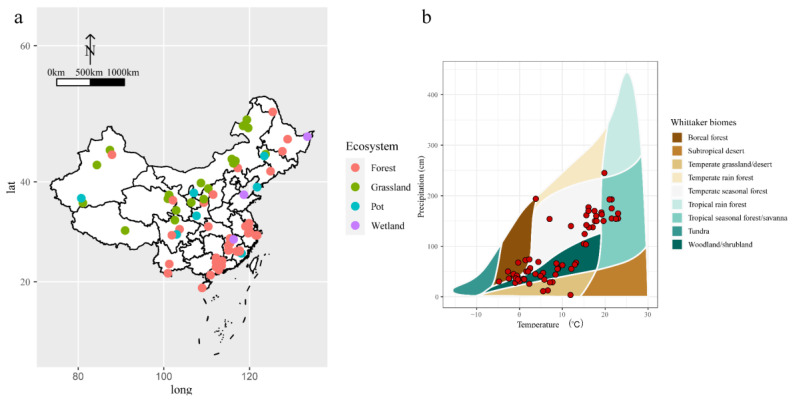
Geographical distribution (**a**) and Whittaker biomes (**b**) of the 75 study sites in China included in the meta-analysis.

Meanwhile, the response of [N], [P], and N:P in plants and soil to N addition did not change with the ecosystem types, suggesting that the changes in ecosystem types were not related to [N], [P], and N:P for the different ecosystem compartments (Table 2). Previous studies have also reported inconsistent N:P responses to N addition across ecosystems and continents [14]. This may be due to the extent of the literature and cases studied per ecosystem and spatial scale. On the other hand, the climatic changes significantly influenced the effects of N addition on [N], [P], and N:P for different ecosystem compartments (Figure 6). Under N addition, our analysis revealed that [N] in leaves and soil increased with increasing MAT, whereas [P] in roots decreased. The ‘Soil Substrate Age N:P hypothesis’ supports these results, suggesting a solid control on leaf N and P due to soil available N and P [47]. The hypothesis indicates that plants will inevitably increase photosynthesis under high-temperature conditions, demanding more N [21,48]. Therefore, high temperatures promote the decomposition and mineralization of soil organic matter, increase the available N in the soil, and then promote nitrogen uptake by plants. Furthermore, under N additions, an increase in MAT increased [N] and [P] in soil (Figure 5b,d). Generally, an increase in temperature accelerates the soil microbial activity and promotes biological N fixation and organic N mineralization rate [49]. Additionally, high temperatures and rainy environments cause N leaching in soil and promote the absorption of available N by roots, supporting an increase in the N:P of the root system (Figure 5e) [21,50].

### 3.3. Implications and Guidelines for Future Work

Several meta-analyses have demonstrated a positive response of [N] and N:P in plants and soil to N addition [2,34]. However, the effects of N addition on [P] in plants and soil and the underlying mechanism remain controversial. One hypothesis suggests that N addition increases available soil P by promoting soil phosphatase activity, promoting plant absorption [28]. However, some empirical studies have found that N addition significantly decreased soil pH, cations, and available [P], which would decrease plant absorption of P [38,39,51]. The present meta-analysis found that increased N addition marginally increased aboveground and underground [N] and N:P stoichiometry. These findings are valuable to forestry production practice. However, research on N deposition is limited to specific ecological compartments or attributes in China, which do not effectively connect the ecosystem’s structure, function, and process.

The interaction between N addition and other climatic factors, such as increased CO_2_ concentration, P addition, drought, global warming, and precipitation pattern, should be considered [6,11]. These interactions may change soil properties and plant community assembly, potentially affecting the stoichiometric ratio of different ecological compartments and the terrestrial N cycle [6,19,46]. Currently, research is limited to the influence of N deposition on the ecological compartments in China. The observations are insufficient, and no discovery has been made on the effects of N addition on litter, which plays a critical role in the N cycle of the entire ecosystem [35]. Therefore, future studies should explore the response of each ecological component to N addition based on global data, which allows us to understand the N cycle in detail [52]. The present meta-analysis will provide a foundation for further studies to elucidate these crucial domains.

## 4. Materials and Methods

### 4.1. Data Collection

The data used for the meta-analysis were collected from peer-reviewed publications published before June 2021 via Web of Science, China National Knowledge database (CNKI), and Science Direct. Search terms such as “N addition” or “N deposition” or “N input and N:P stoichiometric ratio” were employed (Figure S1, Text S1). The following criteria were adopted to include studies for the meta-analysis: (1) Studies were on N addition experiments conducted on field and natural environment under the same abiotic and biotic conditions, in China; (2) If experiments included other treatments (e.g., Phosphorus addition, precipitation reduction, etc.), data were selected only from N addition; (3) The [N] and [P] and the N:P stoichiometry were reported. Here, we only focused on the total nitrogen content obtained by the elemental analyzer and the total phosphorus content obtained by the modified molybdenum-antimony anti-colorimetric method. (4) The mean, and the number of experiments were reported or could be extracted from all experiments and parameters; and (5) The N input rate and experimental duration were reported.

Data on the total N concentrations (TN), total P concentrations (TP), and N:P of leaves, stems, roots, litter, and soil (mass ratio) were collected from each published study. The means of the TN, TP, N:P in the control and N treatment groups and the sampling size were extracted from the published articles. The dataset was extracted as tables or graphs using WebPlotDigitizer 4.2 [53]. Additionally, the ecosystem types and the geographical coordinates of the sites were collected. This study also included pot experiments conducted under ambient precipitation and temperature [42]. For studies conducted in the field, the mean annual temperature (MAT; °C) and the mean annual precipitation (MAP; mm/year) were collected from the cited publication or extracted from the WorldClim database (http://www.wordclim.org/ (accessed on 1 June 2021)) using the latitude and longitude details. Finally, a total of 845 observations from 75 papers, which satisfied the above criteria, were included in the meta-analysis (Figure 6a). Except for tundra and subtropical desert biomes, which were not covered, all others were covered, indicating the representativeness of our data selection (Figure 6b). To control for non-independence in the data due to multiple effect sizes per study and experiment, reference was defined as a random factor.

### 4.2. Data Analysis

Statistical analysis was carried out using R 4.1.0. In this study, the natural logarithm response ratios (ln*RR*) were used to quantify the effects of N addition on the [N], [P], and N:P, using the following equation [50]:

(1) $$\ln RR=\ln(\frac{{\bar{X}}_{t}}{{\bar{X}}_{c}})=\ln{\bar{X}}_{t}-\ln{\bar{X}}_{c}$$

where ${\bar{X}}_{t}$ and ${\bar{X}}_{c}$ represent the mean for the N addition group and the control, respectively [50].

The ln*RR* was weighted based on the number of replicates using the formula:

(2) $$W_{i}=\frac{n_{t}\times n_{c}}{n_{t}+n_{c}}$$

where *n_t_* and *n_c_* represent the number of replicates in the treatment and control groups, respectively [50].

A linear mixed-effect model was used to examine the effects of N addition (*N*, g/m^2^/yr.) and experimental duration (*ED*, days) on [N], [P], and N:P. For the [N], [P], and N:P stoichiometric ratio of the leaves, stems, roots, litter, and soil, we tested whether the ln*RR* differed from zero. If the 95% confidence interval of ln*RR* does not cover 0, it suggests that there is a significant difference in the effect of nitrogen addition on [N], [P], and N:P of plants and soil. Five alternative models, including interaction between the linear and logarithmic terms of *N* and *ED*, were assessed. These models were compared to find those that yielded similar or lower Akaike information criterion (AIC) values (Table S1). The meta-analysis was undertaken subsequently based on Equation (3); the following liner model was used:

(3) $$\ln RR=\beta_{0}+\beta_{1}\ln(N)+\beta_{2}(ED)+\pi_{study}+\varepsilon$$

where *β*_0_ is the mean ln*RR* for the means of the respective predictors, and *β_n_*, π*_study_*, and ε are the coefficients to be calculated, the random effect factor of “study”, and the sampling error, respectively. The predictors’ N input rate (*N*) and experimental duration (*ED*) in Equation (2) were scaled. A restricted maximum likelihood method applied model was performed using the “*lme4*” package [54].

Further, partial linear regression was used to examine the effects of *N* and *ED* on effect size of N addition. The MAT, MAP, and ecosystems were included in Equation (3) to assess the response of ln*RR* to the changes in biogeographic factors ecosystem types. The linear and logarithmic terms of *N* and biogeographic factors or *ED* and biogeographic factors were also considered in the new model, based on Equation (3). Subsequently, similar or lower AIC values were determined without the interaction terms (Tables S2–S4). The histogram and normal quantile–quantile (Q–Q) plot of the residuals were used to check the normality of the model.

## 5. Conclusions

In conclusion, the present meta-analysis confirmed a positive response of [N] and N:P stoichiometry in plants and soil to N addition; however, a marginally negative response was found for [P] in plants and soil. The effects of N addition on [N], [P], and N:P were not consistent with the N input rate and experimental duration. Meanwhile, MAT and MAP regulated the impact of N addition, while the ecosystem type (forest and grassland) had no response to N addition. Thus, this study suggests that the increase in N addition has pronounced effects on the biogeochemical cycling of major elements (N and P) in terrestrial ecosystems, and that the accumulation of soil available N caused by N addition can lead to soil acidification and changes in soil enzyme activity, thereby affecting the N and P cycle of terrestrial ecosystems. Our findings would be necessary for understanding and modelling the effects of N deposition on terrestrial biogeochemical cycles in China.

## Figures and Tables

**Table 1:** *p*-values of N input rate (*N*) and experimental duration (*ED*) on the natural logarithm response ratio (ln*RR*) of [N], [P], and N:P of leaves, litter, roots, soil, and stems. Italicized values indicate a negative correlation.

	N In Put (*N*)			Experimental Duration (*ED*)		
	N	P	N:P	N	P	N:P
Leaf	<0.01	*0.66*	<0.01	0.09	<0.01	*0.02*
Stem	0.34	0.71	0.11	0.68	0.62	0.80
Root	0.15	*0.2*	*0.98*	*0.51*	*0.66*	*0.58*
Litter	<0.01	*0.21*	0.60	0.84	*0.55*	0.61
Soil	0.01	*0.21*	0.45	0.84	0.78	0.93

**Table 2:** Effects of (*p*-value) ecosystem types on natural logarithm response ratio (ln*RR*) of [N], [P], and N:P of different ecosystem compartments leaves, stems, roots, litter, and soil.

	N		P		N:P	
	*df*	*P*	*df*	*P*	*df*	*P*
Leaf	42.74	0.05	42.36	0.40	45.77	0.38
Steam	5.75	0.47	5.57	0.53	4.49	0.85
Root	11.33	0.56	12.50	0.47	11.44	0.66
Litter	7.19	0.20	12.03	0.38	11.32	0.64
Soil	19.82	0.32	19.76	0.47	20.68	0.67

## Data Availability

Database for 854 observations from 75 studies and R code is publicly available at Figshare: https://figshare.com/articles/dataset/Meta_n/16547931 (accessed on 1 June 2021).

## Supplementary Materials

The following supporting information can be downloaded at: https://www.mdpi.com/article/10.3390/plants12112104/s1, Figure S1: Effect sizes (%) of N addition on [N], [P], and N:P for different ecosystem compartments in wetland. The data represent the averages and a 95% CI. Values inside and outside the parentheses represent the number of studies and observations, respectively. Figure S2: Effect sizes (%) of N addition on [N], [P], and N:P for different ecosystem compartments in pots. The data represent the averages and a 95% CI. Values inside and outside the parentheses represent the number of studies and observations, respectively. Figure S3: Workflow diagram showing the procedure for selecting publications. Table S1: The values of Akaike information criterion for five alternative models of [N], [P], N:P. Table S2: The values of Akaike information criterion for four alternative models for biogeographic effects on [N] in different ecosystem compartments. Table S3: The values of Akaike information criterion for four alternative models for biogeographic effects on [P] in different ecosystem compartments. Table S4: The values of Akaike information criterion for four alternative models for biogeographic effects on N:P in different ecosystem compartments. Text S1. Bibliography for studies included in the meta-analysis.
