# Supplementary material for: Response of Plant and Soil N, P, and N:P Stoichiometry to N Addition in China: A Meta-Analysis
Source: Plants (Basel). 2023 May 25;12(11):2104. doi: 10.3390/plants12112104 (PMC10255806; doi:10.3390/plants12112104)
Supplement: Supplementary file 1 [file plants-12-02104-s001.zip › plants-2330554-supplementary.pdf]

plants

MDPI

# Supplementary Material

# Response of plant and soil N, P, and N:P stoichiometry to N addition in China: a meta-analysis

**Table S1.** The values of Akaike information criterion for five alternative models of [N], [P], N:P.

| Stoichiometry | Compartments | Model 1 | Model 2 | Model 3* | Model 4 | Model 5 |
|---|---|---|---|---|---|---|
| [N] | Leaf | -217.227 | -215.241 | -243.249 | -244.507 | -235.448 |
| | Stem | 66.42869 | 66.91511 | 66.42833 | 65.95768 | 72.19932 |
| | Root | -26.2855 | -26.2911 | -27.6801 | -27.6936 | -20.7769 |
| | Litter | 14.48379 | 14.35933 | 14.23491 | 14.38903 | 21.35851 |
| | Soil | -47.0314 | -48.1142 | -47.8415 | -46.7713 | -41.6933 |
| [P] | Leaf | 46.43212 | 47.22489 | 48.41482 | 47.70855 | 54.29541 |
| | Stem | 41.41366 | 41.3569 | 41.23644 | 41.28856 | 47.90919 |
| | Root | 168.1652 | 168.696 | 167.0089 | 166.4888 | 164.5109 |
| | Litter | 106.795 | 106.8576 | 106.164 | 106.1014 | 111.6849 |
| | Soil | 83.16132 | 82.01251 | 80.39408 | 81.54433 | 88.30692 |
| N:P | Leaf | 108.0341 | 106.1289 | 107.1253 | 109.34 | 117.5411 |
| | Stem | 3.804826 | 4.467883 | 3.45609 | 2.925916 | 10.11264 |
| | Root | 59.8189 | 59.8072 | 62.66846 | 62.6519 | 68.42666 |
| | Litter | 118.7249 | 118.3786 | 119.4181 | 119.8077 | 125.5374 |
| | Soil | 211.9405 | 211.6643 | 211.1908 | 211.4552 | 217.2101 |

Model 1: $ln\,R\,R = \beta_0 + \beta_1 \cdot N + \beta_2 \cdot ln\,(ED) + \pi_{study} + \varepsilon$
Model 2: $ln\,R\,R = \beta_0 + \beta_1 \cdot N + \beta_2 \cdot ED + \pi_{study} + \varepsilon$
Model 3: $ln\,R\,R = \beta_0 + \beta_1 \cdot ln(N) + \beta_2 \cdot ED + \pi_{study} + \varepsilon$
Model 4: $ln\,R\,R = \beta_0 + \beta_1 \cdot ln(N) + \beta_2 \cdot ln(ED) + \pi_{study} + \varepsilon$
Model 5: $ln\,R\,R = \beta_0 + \beta_1 \cdot N + \beta_2 \cdot ln(ED) + \beta_3 \cdot \ln\,(C) \times ln(ED) + \pi_{study} + \varepsilon$

**Table S2.** The values of Akaike information criterion for four alternative models for biogeographic effects on [N]. in different ecosystem compartments.

| BF | Compartments | Model 1* | Model 2 | Model 3 | Model 4 |
|---|---|---|---|---|---|
| Ecosystem | Leaf | -237.397 | -232.551 | -228.988 | -224.427 |
| | Stem | 70.57027 | 74.85077 | 71.35619 | 75.56681 |
| | Root | -21.5758 | -10.1992 | -17.51 | -6.11461 |
| | Litter | 21.67222 | 26.06425 | 21.67222 | 26.06425 |
| | Soil | -42.8585 | -26.9799 | -38.9014 | -23.146 |
| MAP | Leaf | -238.053 | -231.043 | -226.505 | -219.406 |
| | Stem | 70.39974 | 76.41697 | 76.37695 | 82.25392 |
| | Root | -25.3444 | -17.0029 | -18.2113 | -9.9138 |
| | Litter | 20.26328 | 25.82372 | 30.0949 | 35.75188 |
| | Soil | -44.4563 | -36.4626 | -33.5941 | -25.62 |
| MAT | Leaf | -242.981 | -235.843 | -234.202 | -226.695 |
| | Stem | 70.90899 | 76.04224 | 76.51896 | 81.40197 |
| | Root | -22.6745 | -14.7857 | -15.0547 | -7.19815 |
| | Litter | 20.7858 | 25.67913 | 30.33726 | 35.2388 |
| | Soil | -43.0667 | -34.9223 | -33.5699 | -25.4249 |

Model 1: $ln\,R\,R = \beta_0 + \beta_1 \cdot ln(N) + \beta_2 \cdot ED + \beta_3 \cdot BF + \pi_{study} + \varepsilon$

Model 2: $ln\,R\,R = \beta_0 + \beta_1 \cdot ln(N) + \beta_2 \cdot ED + \beta_3 \cdot BF + \beta_4 \cdot ln(N) \times BF + \pi_{study} + \varepsilon$

Model 3: $ln\,R\,R = \beta_0 + \beta_1 \cdot ln(N) + \beta_2 \cdot ED + \beta_3 \cdot BF + \beta_4 \cdot ED \times BF + \pi_{study} + \varepsilon$

Model 4: $ln\,R\,R = \beta_0 + \beta_1 \cdot ln(N) + \beta_2 \cdot ED + \beta_3 \cdot BF + \beta_4 \cdot ED \times BF + \beta_5 \cdot ln(ED) \times BF + \pi_{study} + \varepsilon$

BF is biogeographic factor.

**Table S3.** The values of Akaike information criterion for four alternative models for biogeographic effects on [P]. in different ecosystem compartments.

| BF | Compartments | Model 1* | Model 2 | Model 3 | Model 4 |
|---|---|---|---|---|---|
| Ecosystem | Leaf | 59.06462 | 71.20183 | 59.70531 | 72.07189 |
| | Stem | 44.69046 | 48.19602 | 45.4181 | 49.05596 |
| | Root | 171.9546 | 172.2061 | 175.1461 | 175.5973 |
| | Litter | 109.9202 | 112.7688 | 109.9202 | 112.7688 |
| | Soil | 83.64748 | 97.71993 | 85.12673 | 99.09592 |
| MAP | Leaf | 52.58756 | 61.04277 | 52.11274 | 60.628 |
| | Stem | 42.55081 | 47.97038 | 48.33597 | 53.94381 |
| | Root | 169.4878 | 176.2362 | 170.3623 | 177.1401 |
| | Litter | 110.5498 | 115.5011 | 117.7856 | 122.6298 |
| | Soil | 84.68342 | 91.3459 | 93.35462 | 100.0013 |
| MAT | Leaf | 55.28424 | 62.74962 | 59.5419 | 67.13624 |
| | Stem | 42.89964 | 47.22107 | 48.85429 | 53.59513 |
| | Root | 166.4912 | 171.8703 | 172.2557 | 176.9491 |
| | Litter | 109.2465 | 113.443 | 116.7503 | 120.9241 |
| | Soil | 80.04007 | 86.73766 | 88.02004 | 94.76188 |

Model 1: $ln\,R\,R = \beta_0 + \beta_1 \cdot ln(N) + \beta_2 \cdot ED + \beta_3 \cdot BF + \pi_{study} + \varepsilon$

Model 2: $ln\,R\,R = \beta_0 + \beta_1 \cdot ln(N) + \beta_2 \cdot ED + \beta_3 \cdot BF + \beta_4 \cdot ln(N) \times BF + \pi_{study} + \varepsilon$

Model 3: $ln\,R\,R = \beta_0 + \beta_1 \cdot ln(N) + \beta_2 \cdot ED + \beta_3 \cdot BF + \beta_4 \cdot ED \times BF + \pi_{study} + \varepsilon$

Model 4: $ln\,R\,R = \beta_0 + \beta_1 \cdot ln(N) + \beta_2 \cdot ED + \beta_3 \cdot BF + \beta_4 \cdot ED \times BF + \beta_5 \cdot ln(ED) \times BF + \pi_{study} + \varepsilon$

BF is biogeographic factor.

**Table S4.** The values of Akaike information criterion for four alternative models for biogeographic effects on N:P. in different ecosystem compartments.

| BF | Compartments | Model 1* | Model 2 | Model 3 | Model 4 |
|---|---|---|---|---|---|
| Ecosystem | Leaf | 116.8842 | 123.519 | 122.7952 | 129.7726 |
| | Stem | 11.76276 | 17.2789 | 14.01156 | 19.46015 |
| | Root | 68.08816 | 78.44004 | 71.49645 | 81.82536 |
| | Litter | 123.0649 | 126.1297 | 123.0649 | 126.1297 |
| | Soil | 218.0705 | 229.1263 | 220.6201 | 231.6887 |
| MAP | Leaf | 113.8121 | 120.4125 | 125.3686 | 132.0419 |
| | Stem | 5.927994 | 12.96481 | 13.07851 | 19.23225 |
| | Root | 61.97878 | 64.71476 | -18.2113 | 69.96128 |
| | Litter | 124.1693 | 128.9971 | 128.4671 | 133.4218 |
| | Soil | 215.4336 | 221.1136 | 223.042 | 228.8875 |
| MAT | Leaf | 110.6094 | 117.9968 | 124.7684 | 132.1511 |
| | Stem | 9.059454 | 15.4531 | 16.2843 | 22.30091 |
| | Root | 66.34015 | 70.08223 | 72.14685 | 75.61861 |
| | Litter | 124.1598 | 127.6732 | 127.377 | 130.9315 |
| | Soil | 214.0091 | 220.0158 | 221.3801 | 227.4778 |

Model 1: $ln\,R\,R = \beta_0 + \beta_1 \cdot ln(N) + \beta_2 \cdot ED + \beta_3 \cdot BF + \pi_{study} + \varepsilon$
Model 2: $ln\,R\,R = \beta_0 + \beta_1 \cdot ln(N) + \beta_2 \cdot ED + \beta_3 \cdot BF + \beta_4 \cdot ln(N) \times BF + \pi_{study} + \varepsilon$
Model 3: $ln\,R\,R = \beta_0 + \beta_1 \cdot ln(N) + \beta_2 \cdot ED + \beta_3 \cdot BF + \beta_4 \cdot ED \times BF + \pi_{study} + \varepsilon$
Model 4: $ln\,R\,R = \beta_0 + \beta_1 \cdot ln(N) + \beta_2 \cdot ED + \beta_3 \cdot BF + \beta_4 \cdot ED \times BF + \beta_5 \cdot ln(ED) \times BF + \pi_{study} + \varepsilon$
BF is biogeographic factor.

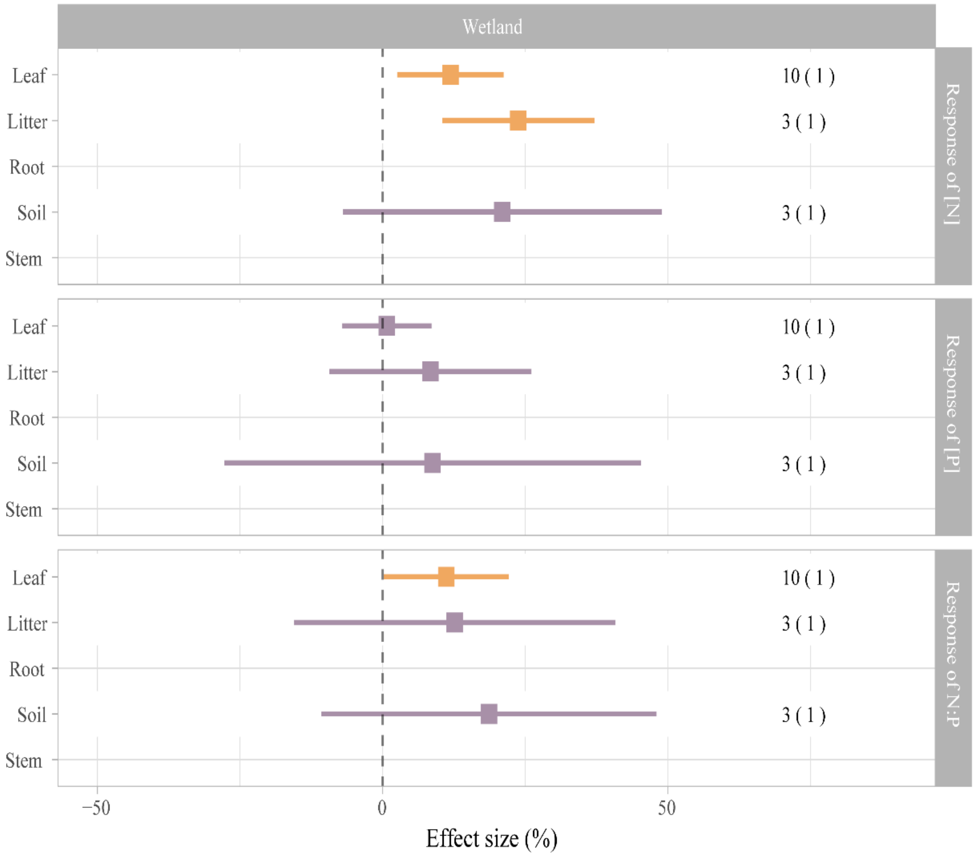


**Figure S1.** Effect sizes (%) of N addition on [N], [P], and N:P for different ecosystem compartments in wetland. The data represent the averages and a 95% CI. Values inside and outside the parentheses represent the number of studies and observations, respectively.

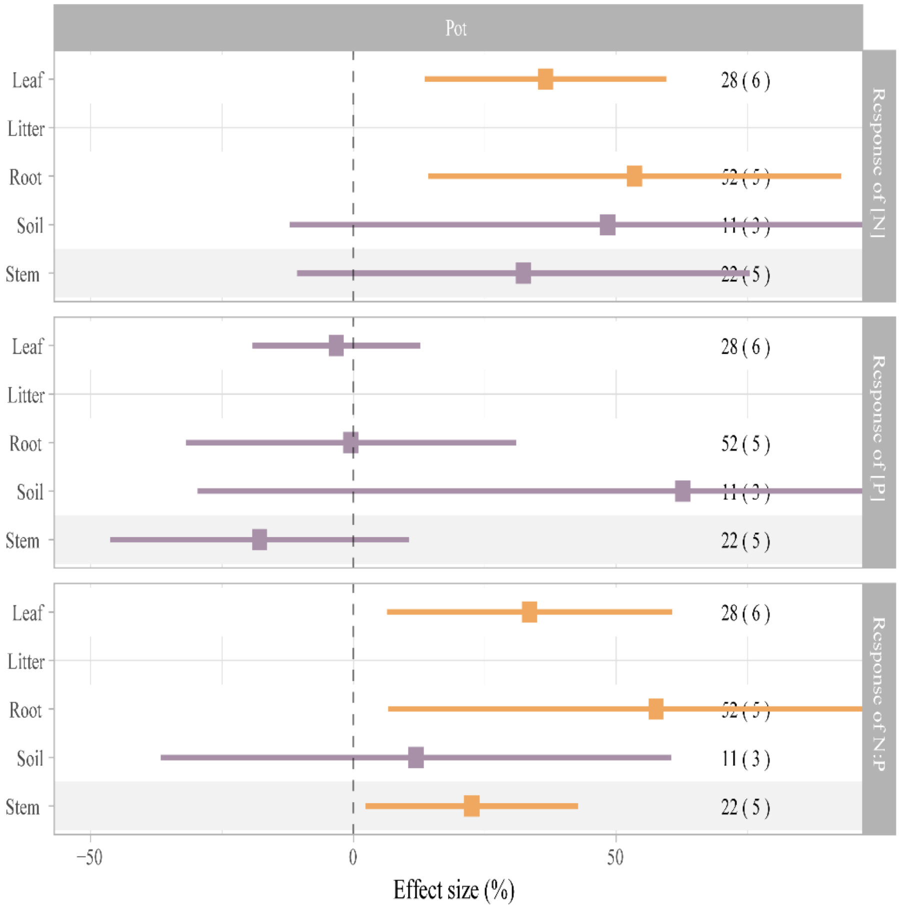


**Figure S2.** Effect sizes (%) of N addition on [N], [P], and N:P for different ecosystem compartments in Pot. The data represent the averages and a 95% CI. Values inside and outside the parentheses represent the number of studies and observations, respectively.

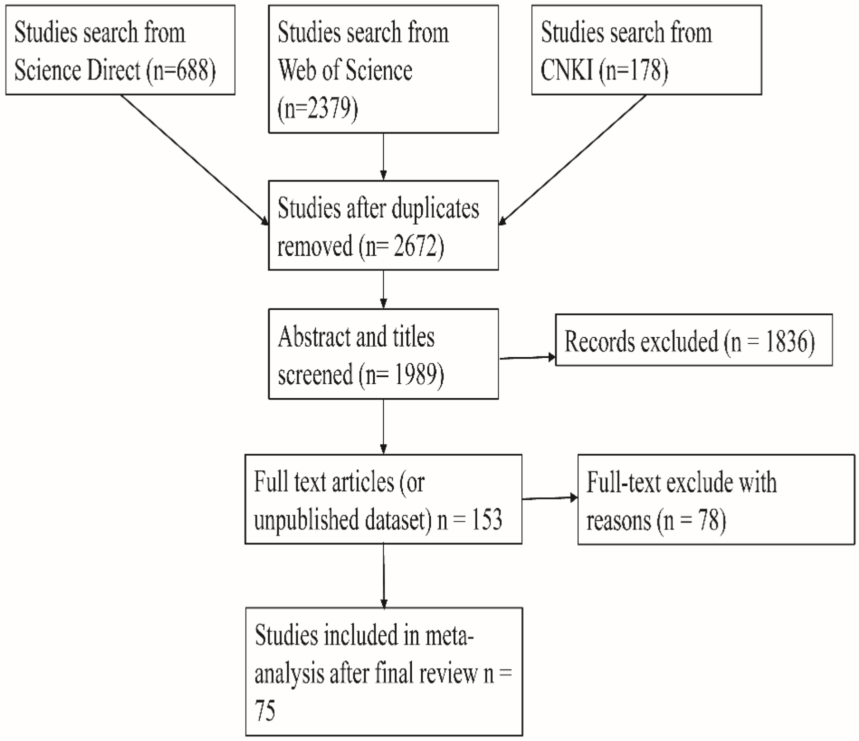


**Figure S3.** Workflow diagram showing the procedure for selecting publications.

**Text S1.** Bibliography for studies included in the meta-analysis.

Ai, Z.-M., S. Xue, G.-L. Wang, and G.-B. Liu. 2017. Responses of Non-structural Carbohydrates and C:N:P Stoichiometry of Bothriochloa ischaemum to Nitrogen Addition on the Loess Plateau, China. Journal of Plant Growth Regulation **36**:714-722.

Chen, F.-S., K. J. Niklas, Y. Liu, X.-M. Fang, S.-Z. Wan, and H. Wang. 2015. Nitrogen and phosphorus additions alter nutrient dynamics but not resorption efficiencies of Chinese fir leaves and twigs differing in age. Tree Physiology **35**:1106-1117.

Chen, F. S., X. Feng, and C. Liang. 2012. Endogenous versus exogenous nutrient affects C, N, and P dynamics in decomposing litters in mid-subtropical forests of China. Ecological Research **27**:923-932.

Chen, L. L., Z. Y. Yuan, T. R. Lock, and R. L. Kallenbach. 2021. The production and nutrients in the fine roots of fertilized Pinus tabulaeformis plantation forests in the Loess Plateau in China. Journal of Forest Research.

Chen, Y., W. Wang, R. Huang, H. Luo, y. Li, Y. Li, Q. Zhao, and Q. Mo. 2018. Response of foliar physiological characteristic within two understory plant species to chronic nitrogen and phosphorus addition in a secondary tropical forest. Chinese Journal of Applied Ecology **25**:626-633.

Cui, X. 2018. Effects of enhanced precipitation temperature and nitrogen addition on nitrogen fate and plant stoichiometry in temperate desert ecosystem in XinJiang®. China Agricultural University.

Fu, Y., D. Tian, S. Niu, and K. Zhao. 2020. Effects of nitrogen, phosphorus addition and drought on leaf stoichiometry in dominant species of alpine meadow. Journal of Beijing Forestry University **42**:119-127.

Guo J B, Zhao Guo Q, Jia SG, Dong JF, Chen L., Wang SP. 2020. Comprehensive evaluation of effects of fertilization on grassland quality index and soil properties in alpine steppe. Acta Prataculturae Simica. 29(9): 85-93.

Han, X., S. A. Sistla, Y.-H. Zhang, X.-T. Lu, and X.-G. Han. 2014. Hierarchical responses of plant stoichiometry to nitrogen deposition and mowing in a temperate steppe. Plant and Soil **382**:175-187.

Han, X., A. Tsunekawa, M. Tsubo, and H. Shao. 2013. Responses of plant-soil properties to increasing N deposition and implications for large-scale eco-restoration in the semiarid grassland of the northern Loess Plateau, China. Ecological Engineering **60**:1-9.

Hou, S.-L., J.-X. Yin, J.-J. Yang, H.-W. Wei, G.-J. Yang, Y.-Y. Hu, X.-G. Han, and X.-T. Lu. 2017. Consistent responses of litter stoichiometry to N addition across different biological organization levels in a semi-arid grassland. Plant and Soil **421**:191-202.

Hu, X. Y., and Z. G. Sun. 2021. Effects of exogenous nitrogen import on variations of nutrient in decomposing litters of Suaeda salsa in coastal marsh of the Yellow River estuary, China. Environmental Science and Pollution Research **28**:33165-33180.

Hu, Y.-Y., H.-W. Wei, Z.-W. Zhang, S.-L. Hou, J.-J. Yang, J.-F. Wang, and X.-T. Lu. 2020. Changes of plant community composition instead of soil nutrient status drive the legacy effects of historical nitrogen deposition on plant community N:P stoichiometry. Plant and Soil **453**:503-513.

Huang, J., P. Wang, Y. Niu, H. Yu, F. Ma, G. Xiao, and X. Xu. 2018. Changes in C:N:P stoichiometry modify N and P conservation strategies of a desert steppe species Glycyrrhiza uralensis. Scientific Reports **8**.

Huang, W., G. Zhou, J. Liu, D. Zhang, Z. Xu, and S. Liu. 2012. Effects of elevated carbon dioxide and nitrogen addition on foliar stoichiometry of nitrogen and phosphorus of five tree species in subtropical model forest ecosystems. Environmental Pollution **168**:113-120.

Jin, X., L. Yang, X. Yang, Q. Guan, Z. Ma, J. Pan, X. Jiang, and H. Hou. 2020. Effects of N and P fertilization on the biomass and ecological stoichiometric characteristics ofAgropyron michnoiin sandy grasslands. Chemistry and Ecology **36**:938-952.

Jing, H., H. Zhou, G. Wang, S. Xue, G. Liu, and M. Duan. 2017. Nitrogen Addition Changes the Stoichiometry and Growth Rate of Different Organs in Pinus tabuliformis Seedlings. Frontiers in Plant Science **8**.

Kou, L., W. Chen, L. Jiang, X. Dai, X. Fu, H. Wang, and S. Li. 2018. Simulated nitrogen deposition affects stoichiometry of multiple

elements in resource-acquiring plant organs in a seasonally dry subtropical forest. Science of the Total Environment **624**:611-620.

Li, D. 2020. Effects of nitrogen and phosphorus addition on community and stoichiometric characteristics of carbon, nitrogen and phosphorus in grassland ecosystem of northern China. University of Chinese Academy of Sciences.

Li, J., C. Yang, X. Liu, and X. Shao. 2019a. Inconsistent stoichiometry response of grasses and forbs to nitrogen and water additions in an alpine meadow of the Qinghai-Tibet Plateau. Agriculture Ecosystems & Environment **279**:178-186.

Li, J. H., R. Zhang, B. H. Cheng, L. F. Ye, W. J. Li, and X. M. Shi. 2021a. Effects of nitrogen and phosphorus additions on decomposition and accumulation of soil organic carbon in alpine meadows on the Tibetan Plateau. Land Degradation & Development **32**:1467-1477.

Li, M., B. Jin, Q. Zhong, Y. Mam, H. Lu, B. Guo, Y. Zheng, and D. Cheng. 2016. Effect of nitrogen and phosphorus fertilization on leaf N and P stoichiometric ffect of nitrogen and phosphorus fertilization on leaf N and P stoichiometric characteristics of haracteristics of Machilus pauhoi achilus pauhoi seedlings. Chinese Journal of Applied and environmental Biology **022**:285-291.

Li, R., Y. Lu, Y. Wang, and F. Wan. 2019b. Effects of N addition on C, N and P stoichiometry and soil enzyme activities in Cupressuslusitanica Mill. plantation. Chinese Journal of Ecology **38**:78-87.

Li, S., F. Wang, M. Chen, Z. Liu, L. Zhou, J. Deng, C. Dong, G. Bao, T. Bai, Z. Li, H. Guo, Y. Wang, Y. Qiu, and S. Hu. 2020. Mowing alters nitrogen effects on the community-level plant stoichiometry through shifting plant functional groups in a semi-arid grassland. Environmental Research Letters **15**.

Li, S., Y. Zhang, and J. Guo. 2021b. Effects of simulated nitrogen deposition on ecological stoichiometric characteristics of leaf litter in Larix principis-rupprechtii stand. Science of Soil and Water Conservation **19**:7.

Liao, K., F. Shen, W. Liu, Q. Meng, H. Tong, G. Chen, J. Xu, and H. Fan. 2020. C，N and P stoichiometric characteristics of litterfall and soil in a Chinese fir plantation under

long-term nitrogen deposition. Guihaia **40**:1551-1562.

Liu, G. C., Y. J. Xing, Q. G. Wang, L. Wang, Y. Feng, Z. W. Yin, X. C. Wang, and T. Liu. 2021a. Long-term nitrogen addition regulates root nutrient capture and leaf nutrient resorption in Larix gmelinii in a boreal forest. European Journal of Forest Research **140**:763-776.

Liu, J., W. Liu, X.-E. Long, Y. Chen, T. Huang, J. Huo, L. Duan, and X. Wang. 2020. Effects of nitrogen addition on C:N:P stoichiometry in moss crust-soil continuum in the N-limited Gurbantunggut Desert, Northwest China. European Journal of Soil Biology **98**.

Liu, M. H., Y. K. Shen, Q. Li, W. F. Xiao, and X. Z. Song. 2021b. Arbuscular mycorrhizal fungal colonization and soil pH induced by nitrogen and phosphorus additions affects leaf C:N:P stoichiometry in Chinese fir (Cunninghamia lanceolata) forests. Plant and Soil **461**:421-440.

Liu, Y., R. Xu, X. Xu, D. Wei, Y. Wang, and Y. Wang. 2013a. Plant and soil responses of an alpine steppe on the Tibetan Plateau to multi-level nitrogen addition. Plant and Soil **373**:515-529.

Liu, Y., J. Zhang, Y. Chen, L. Chen, and Q. Liu. 2013b. Effect of nitrogen and phosphorus fertilization on biomass allocation and C:N:P stoichiometric characteristics of Eucalyptus grandis seedlings. Chinese Journal of Plant Ecology:37-45.

Long, M., H.-H. Wu, M. D. Smith, K. J. La Pierre, X.-T. Lu, H.-Y. Zhang, X.-G. Han, and Q. Yu. 2016. Nitrogen deposition promotes phosphorus uptake of plants in a semi-arid temperate grassland. Plant and Soil **408**:475-484.

Lu, X.-T., D.-L. Kong, Q.-M. Pan, M. E. Simmons, and X.-G. Han. 2012. Nitrogen and water availability interact to affect leaf stoichiometry in a semi-arid grassland. Oecologia **168**:301-310.

Mao, Q., X. Lu, H. Mo, P. Gundersen, and J. Mo. 2018. Effects of simulated N deposition on foliar nutrient status, N metabolism and photosynthetic capacity of three dominant understory plant species in a mature tropical forest. Science of the Total

Environment **610**:555-562.

Mo, Q., W. Wang, Y. Chen, Z. Peng, and Q. Zhou. 2020. Response of foliar functional traits to experimental N and P addition among overstory and understory species in a tropical secondary forest. Global Ecology and Conservation **23**.

Niu, J., T. P. Lu, Y. J. Lin, and W. X. Zhang. 2020. Effects of nitrogen addition on the characteristics of foliar and soil ecological stoichiometry in xishuangbanna tropical rainforest, southwest China Journal of Tropical Forest Science **32**:1-7.

Pan, Y., K. Wang, Y. Song, Y. Zhang, and X. Zheng. 2021. Short-term responses of litter nutrient release and soil ecological stoichiometry to simulated nitrogen deposition in Pinus armandii forest in central Yunnan, China Ecology and Environmental Sciences **30**:492-502.

Shen, F., Y. Li, W. Liu, H. Duan, H. Fan, L. Hu, and q. Meng. 2019. Responses of nitrogen and phosphorus resorption from leaves and branches to long-term nitrogen deposition in a Chinese fir plantation Chinese Journal of Plant Ecology **42**.

Shi, B., X. Ling, H. Cui, W. Song, Y. Gao, and W. Sun. 2020. Response of nutrient resorption ofLeymus chinensisto nitrogen and phosphorus addition in a meadow steppe of northeast China. Plant Biology **22**:1123-1132.

Song, X., H. Gu, M. Wang, G. Zhou, and Q. Li. 2016. Management practices regulate the response of Moso bamboo foliar stoichiometry to nitrogen deposition. Scientific Reports **6**.

Su, Y., Y. Luo, F. Geng, W. Han, Y. Zhu, K. Li, and X. Liu. 2019. Response of Stoichiometric Characteristics of Nitrogen and Phosphorus in Plant Leaves in an Alpine Grasslands to Nitrogen Deposition

in the Tianshan Mountains. Arid Zone Reeearch **36**:430-436.

Tie, L. H., R. Fu, J. Penuelas, J. Sardans, S. B. Zhang, S. X. Zhou, J. X. Hu, and C. D. Huang. 2020a. The Additions of Nitrogen and Sulfur Synergistically Decrease the Release of Carbon and Nitrogen from Litter in a Subtropical Forest. Forests **11**.

Tie, L. H., S. B. Zhang, J. Penuelas, J. Sardans, S. X. Zhou, J. X. Hu, and C. D. Huang. 2020b. Responses of soil C, N, and P stoichiometric ratios to N and S additions in a subtropical evergreen broad-leaved forest. Geoderma **379**.

Wan, S. Z., G. S. Yang, and R. Mao. 2020. Responses of leaf nitrogen and phosphorus allocation patterns to nutrient additions in a temperate freshwater wetland. Ecological Indicators **110**.

Wang, F., F. Chen, G. G. Wang, R. Mao, X. Fang, H. Wang, and W. Bu. 2019a. Effects of Experimental Nitrogen Addition on Nutrients and Nonstructural Carbohydrates of Dominant Understory Plants in a Chinese Fir Plantation. Forests **10**.

Wang, H.-Y., Z.-W. Wang, R. Ding, S.-L. Hou, G.-J. Yang, X.-T. Lu, and X.-G. Han. 2018a. The impacts of nitrogen deposition on community N:P stoichiometry do not depend on phosphorus availability in a temperate meadow steppe. Environmental Pollution **242**:82-89.

Wang, H., R. Ding, Z. Wang, and F. Yang. 2020a. Effects of nitrogen and phosphorus addition on C:N:P ecological stoichiometry in leaves and roots of different canopy species in Hulunbuir grassland. Acta Prataculturae Sinica **29**.

Wang, M., C. Wang, L. Yang, and H. Guo. 2019b. Impacts of short-term nitrogen addition on the thallus nitrogen and phosphorus balance of the dominant epiphytic lichens in the Shennongjia mountains, China. Journal of Plant Ecology **12**:751-758.

Wang, X., W. Luo, Q. Yu, C. Tan, Z. Xu, and M. Li. 2014. Effects of nutrient addition on nitrogen，phosphorus and non-structural carbohydrates

concentrations in leaves of dominant plant species in a semiarid steppe. Chinese Journal of Ecology **33**:1795-1802.

Wang, X. G., Wuyunna, C. A. Busso, Y. T. Song, F. J. Zhang, and G. W. Huo. 2018b. Responses of C:N:P stoichiometry of plants from a Hulunbuir grassland to salt stress, drought and nitrogen addition. Phyton-International Journal of Experimental Botany **87**:123-132.

Wang, Y., X. Fu, H. Wang, X. Dai, L. Kou, and X. Fang. 2021. Effects of nitrogen and phosphorus additions on microbial community structure and enzyme activity in root and leaf decris of cunninghamia lanceolata. Acta Ecologica Sinica **41**.

Wang, Y., X. Hu, F. Wang, Y. Zhang, and S. Chen. 2016. Effects of Nitrogen and Phosphorus Fertilization on Nutrient Dynamics and Stoichiometric Ratios of Three-Understory Plants in Chinese Fir Plantation. Acta Agriculturae Universitatis Jiangxiensis

38:304-311.

Wang, Y., M. Zheng, S. Wang, J. Mao, and J. Mo. 2020b. Effects of Long-term Nitrogen and Phosphorus Additions on Soil Enzyme Activities Related N and P Cycle in Two Plantations in South China. Journal of Tropical and Subtropical Botany **29**:240-250.

Wang, Z. 2020. Response of leaves, stems and roots stoichiometric characteristics of Acer mono and Pinus tabulaeformis from different provenances to simulated nitrogen deposition BeiJing Forestry University.

Wen, M., Q. Hu, W. Yang, Q. Wu, and B. Yao. 2021. Effects of nitrogen and phosphorus addition on soil nutrients and plant biomass in a typical Poyang Lake marshland. Chinese Journal of Ecology.

Wu, X. Y., X. H. Du, S. Y. Fang, J. Y. Kang, Z. C. Xia, and Q. X. Guo. 2020. Impacts of competition and nitrogen addition on plant stoichiometry and non-structural carbohydrates in two larch species. Journal of Forestry Research.

Xie, Y., M. Lin, H. Xu, Z. Wang, and Y. Li. 2019. Growth performance of Ormosia balansae seedlings and their feedbacks on the soil nutrient under varied soil nitrogen and phosphorus addition conditions. Ecological Science **038**:56-66.

Yan, T., T. Qu, H. Song, P. Ciais, S. Piao, Z. Sun, and H. Zeng. 2018. Contrasting effects of N addition on the N and P status of understory vegetation in plantations of sapling and mature Larix principis-rupprechtii. Journal of Plant Ecology **11**:843-852.

Yang, D., L. Song, and G. Jin. 2019. The soil C:N:P stoichiometry is more sensitive than the leaf C:N:P stoichiometry to nitrogen addition: a four-year nitrogen addition experiment in a Pinus koraiensis plantation. Plant and Soil **442**:183-198.

Yang, H. 2018. Effects of nitrogen and phosphorus addition on leaf nutrient characteristics in a subtropical forest. Trees-Structure and Function **32**:383-391.

Yang, L., G. Wang, y. Yang, and y. Yang. 2012. Responses of leaf functional traits and nitrogen and phosphorus stoichiometry in Abies fabiri seedlings in Gongga Mountain to simulated nitrogen deposition. Chinese Journal of Ecology **31**:44-50.

Yang, Q., Q. Li, J. B. Zhang, W. F. Xiao, and X. Z. Song. 2021. Phosphorus Addition Increases Aboveground Biomass but Does Not Change N:P Stoichiometry of Chinese fir (Cunninghamia lanceolata) Seedlings under Nitrogen Deposition. Polish Journal of Environmental Studies **30**:1421-1431.

Yao, J., J. Lv, W. Yu, J. Zhang, Z. Lei, q. Li, and X. Song. 2018. Effects of nitrogen deposition and management intensity on stoichiometry of leaf litter in

Moso bamboo forest. Chinese Journal of Applied Ecology **29**:467-473.

Yue, Z., X. Li, L. Li, L. Lin, B. Liu, and F. Zeng. 2020. Responses of soil, microbes and plant ecological stoichiometric characteristics to nitrogen addition in an alpine grassland of Kunlun Mountain. Ecological Science **39**:1-8.

Zhai, J. 2020. Effects of Nitrogen and Phosphorus Addition on Soil Microbes and Stoichiometric Characteristic of Alpine Meadow in Qinghai-Tibet Plateau University of Chinese Academy of Sciences

Zhang, N., R. Guo, P. Song, J. Guo, and Y. Gao. 2013. Effects of warming and nitrogen deposition on the coupling mechanism between soil nitrogen and phosphorus in Songnen Meadow Steppe, northeastern China. Soil Biology & Biochemistry **65**:96-104.

Zhang, Q., J. Xie, M. Lyu, D. Xiong, J. Wang, Y. Chen, Y. Li, M. Wang, and Y. Yang. 2017. Short-term effects of soil warming and nitrogen addition on the N:P stoichiometry of Cunninghamia lanceolata in subtropical regions. Plant and Soil **411**:395-407.

Zhang, R., Y. Zhao, J. Lin, Y. Hu, H. Hanninen, and J. Wu. 2019a. Biochar application alleviates unbalanced nutrient uptake caused by N deposition in Torreya grandis trees and seedlings. Forest Ecology and Management **432**:319-326.

Zhang, X., T. Lu, H. Sun, and H. Zhao. 2019b. Effects of Nitrogen and Phosphorus Addition on Nutrient Stoichiometry and resorption of Fraxinus mandshurica. Forest Engineering **35**:16-21.

Zhang, Z., A. Tariq, F. Zeng, X. Chai, and C. Graciano. 2021. Involvement of soluble proteins in growth and metabolic adjustments of drought-stressed Calligonum mongolicum seedlings under nitrogen addition. Plant Biology **23**:32-43.

Zheng, S. 2020. Effects of nitrogen and phosphorus addition on stoichiometry of Leymus chinensis-soil-rhizosphere microbe. Northeast Normal University.

Zheng, Z., M. Mamuti, H. Liu, Y. Shu, S. Hu, X. Wang, B. Li, L. Lin, and X. Li. 2017. Effects of nutrient additions on litter decomposition

regulated by phosphorus-induced changes in litter chemistry in a subtropical forest, China. Forest Ecology and Management **400**:123-128.

Zheng, Z. M., J. Lu, Y. Q. Su, Q. S. Yang, Y. H. Lin, H. M. Liu, J. Yang, H. Huang, and X. H. Wang. 2020. Differential effects of N and P additions on foliar stoichiometry between species and community levels in a subtropical forest in eastern China. Ecological Indicators **117**.

Zhu, W., J. Wang, Z. Zhang, F. Ren, L. Chen, and J.-S. He. 2016. Changes in litter quality induced by nutrient addition alter litter decomposition in an alpine meadow on the Qinghai-Tibet Plateau. Scientific Reports **6**.

Zhuang, L. Y., Q. Liu, Z. Y. Liang, C. M. You, B. Tan, L. Zhang, R. Yin, K. J. Yang, R. Bol, and Z. F. Xu. 2020. Nitrogen Additions Retard Nutrient Release from Two Contrasting Foliar Litters in a Subtropical Forest, Southwest China. Forests **11**.
